# Comparative effectiveness and safety of adjuvant trastuzumab plus pertuzumab versus trastuzumab emtansine in HER2-positive breast cancer with residual disease after neoadjuvant therapy: a real-world retrospective study

**DOI:** 10.3389/fonc.2026.1852055

**Published:** 2026-06-26

**Authors:** Zhenghua Liu, Xinyu Wang, Jiayan Gai, Chen Wang, Xinlu Yi, Yufei Liu, Tao Zhou

**Affiliations:** 1 Breast Disease Center, The Fourth Hospital of Hebei Medical University, Shijiazhuang, Hebei, China; 2 Hebei Medical University, Shijiazhuang, Hebei, China

**Keywords:** HER2-positive breast cancer, pertuzumab, real-world study, residual disease, trastuzumab emtansine

## Abstract

**Purpose:**

To compare the effectiveness, safety, and tolerability of adjuvant trastuzumab plus pertuzumab (HP) versus trastuzumab emtansine (T-DM1) in patients with HER2-positive breast cancer with residual invasive disease after neoadjuvant therapy, and to perform exploratory analyses of outcomes in clinically favorable subgroups.

**Materials and methods:**

Patients with HER2-positive breast cancer and residual invasive disease after NAT, enrolled between 2020 and 2024, were included. Propensity score matching (1:1) was applied to adjust for baseline characteristic differences. Kaplan-Meier survival analysis and Cox proportional hazards models were used to compare survival outcomes (iDFS, RFS, OS) between the two groups. Additionally, the incidence of adverse events and treatment adherence were compared.

**Results:**

A total of 272 patients were analyzed, with 134 remaining after propensity score matching. After a median follow-up of 37.7 months, no statistically significant differences in short-term survival outcomes were detected between the two groups. Grade 3 or higher adverse events occurred more frequently in the T-DM1 group, particularly thrombocytopenia. Treatment interruption or regimen modification occurred in 22.7% of patients in the T-DM1 group and 2.2% in the HP group.

**Conclusion:**

In HER2-positive breast cancer patients with residual invasive disease after NAT, no statistically significant difference in short-term recurrence or survival outcomes was detected between adjuvant HP and T-DM1, while HP was associated with a more favorable safety and tolerability profile. These findings should be interpreted as complementary real-world evidence in the contemporary dual-blockade era and as hypothesis-generating support for future risk-adapted adjuvant strategies.

## Introduction

HER2-positive breast cancer comprises approximately 20%–25% of all breast cancer cases. It is associated with aggressive tumor behavior and a high risk of recurrence and distant metastasis, particularly to high-risk sites such as the central nervous system (1–3). The introduction of HER2-targeted therapies has significantly reshaped the treatment landscape for this subtype, leading to substantial improvements in patient survival outcomes.

The phase III KATHERINE trial (NCT01772472) established trastuzumab emtansine (T-DM1) as the standard treatment for patients with HER2-positive breast cancer who did not achieve pathological complete response (pCR) after neoadjuvant therapy (NAT). In that trial, T-DM1 reduced the risk of invasive disease recurrence or death by 50% compared with trastuzumab alone (4). However, the KATHERINE trial was conducted between 2013 and 2015, when dual HER2 blockade was not routinely used, and up to 80% of enrolled patients had received trastuzumab monotherapy during the neoadjuvant phase. In current clinical practice, trastuzumab plus pertuzumab (HP) is widely used in both neoadjuvant and adjuvant treatment settings (5, 6). Therefore, although T-DM1 remains the standard post-NAT therapy for residual disease, the real-world comparative outcomes of adjuvant HP and T-DM1 in patients treated in the contemporary dual-blockade era remain unclear.

In addition to potential differences in efficacy, safety and patient compliance are key factors in clinical decision-making. In the KATHERINE trial, 18% of patients in the T-DM1 arm discontinued treatment due to adverse events (AEs), with an additional 14.3% requiring dose reductions. In contrast, the treatment discontinuation rate in the trastuzumab arm was only 2.1% (4). Furthermore, a large-scale real-world cohort study from France showed that patients treated with T-DM1 for early-stage breast cancer had increased risks of hospitalization for hematologic disorders and thrombocytopenia compared with incident breast cancer patients in the general population (7). As antibody-drug conjugates (ADCs) are increasingly utilized in early-stage breast cancer, managing cumulative toxicity presents significant challenges. For instance, the incidence of Grade ≥ 3 AEs with T-DXd is up to 51.9%, with a 9.6% risk of interstitial lung disease (ILD) (8). In contrast, dual HER2 blockade with HP has demonstrated favorable safety and long-term tolerability in the adjuvant setting (6). Moreover, fixed-dose subcutaneous formulations of pertuzumab and trastuzumab have shown comparable pharmacokinetics, efficacy, and safety to intravenous administration and may improve treatment convenience (9).

Although pCR is a key surrogate endpoint, its ability to predict long-term benefit is limited, particularly in the HR-positive/HER2-positive population (10). HR-positive/HER2-positive tumors generally achieve lower pCR rates, however, this does not necessarily correlate with significantly poorer long-term outcomes. Clinical prognosis in this subgroup is influenced by various factors, including residual tumor burden (RCB), intrinsic tumor biology, and the use of postoperative endocrine therapies (11–14). Moreover, considerable heterogeneity exists within the non-pCR population after NAT. A large-scale study demonstrated that RCB grading further differentiates subgroups with varying recurrence risks, with patients in RCB grades I–II exhibiting significantly better long-term survival compared with those in RCB grade III (15, 16). In addition, emerging evidence suggests that HER2DX may further refine molecular risk stratification in early-stage HER2-positive breast cancer by integrating tumor biology with clinical risk factors (17–19). Therefore, evaluating real-world treatment outcomes within biologically and pathologically defined subgroups may help refine future risk stratification and prospective trial design.

This study primarily compared the effectiveness of HP and T-DM1 in HER2-positive breast cancer patients who did not achieve pCR after NAT. In addition, it assessed differences in safety, tolerability, and treatment adherence between the two regimens in a real-world setting. We also performed exploratory subgroup analyses in clinically favorable populations, defined by biological and postoperative pathological features such as HR-positive/HER2-positive status or RCB I–II. This study was not designed to challenge the standard role of T-DM1; rather, it aimed to provide complementary real-world evidence from the contemporary dual-blockade era and to generate hypotheses for future risk-adapted adjuvant strategies.

## Materials and methods

### Study design and population

This single-center, retrospective, observational study was conducted at the Fourth Hospital of Hebei Medical University and included patients with HER2-positive breast cancer who underwent surgery following NAT between January 2020 and December 2024. The inclusion criteria were: (1) age > 18 years; (2) HER2-positive status confirmed by core needle biopsy (immunohistochemistry [IHC] 3+ or fluorescence *in situ* hybridization [FISH]-positive); (3) receipt of at least 4 cycles of HER2-targeted NAT; (4) Eastern Cooperative Oncology Group (ECOG) performance status 0–1. Exclusion criteria included: (1) pregnancy-associated breast cancer; (2) disease progression during NAT; (3) failure to achieve R0 resection; (4) history of other malignancies; (5) recurrent or bilateral breast cancer; (6) incomplete clinical and pathological data. The study was approved by the Ethics Committee of the Fourth Hospital of Hebei Medical University (Approval Number: 2025KS144). Written informed consent was waived due to the use of de-identified data and minimal risk.

### Data collection

Clinical and pathological data were extracted from the electronic medical record system. Baseline characteristics included demographic information (age, sex, menopausal status, family history of malignancy), disease-related variables (American Joint Committee on Cancer [AJCC] stage prior to NAT, tumor size and location, molecular subtype, surgical procedure, pathological findings, AJCC stage post-NAT, pathological features of residual tumor, Miller-Payne [MP] grade, and RCB grade), and treatment regimens (chemotherapy, HER2-targeted therapy). Luminal B (HER2-positive) breast cancer was defined as tumors expressing ER/PR ≥ 1% and HER2-positive status, while tumors with both ER/PR <1% and HER2-positive status were categorized as purely HER2-positive. pCR was defined as the absence of residual invasive carcinoma in both the breast and axillary lymph nodes after NAT (ypT0/is, ypN0). Breast pathological complete response (bpCR) referred to the absence of residual invasive carcinoma in the breast (ypT0/is). Patients who did not achieve pCR were categorized as non-pCR. RCB grading was independently assessed by a dedicated breast pathologist using the validated MD Anderson RCB index (20).

During postoperative adjuvant therapy, patients received either HP (trastuzumab 6 mg/kg plus pertuzumab 420 mg every 3 weeks) or T-DM1 (3.6 mg/kg every 3 weeks) for a planned duration of 1 year. Patients with incomplete or unclear postoperative treatment data were excluded. Information on postoperative regimens and AEs was obtained from the electronic medical record system and supplemented by telephone follow-up.

### Endpoints

Study endpoints were defined according to the Standardized Definitions for Efficacy End Points (STEEP) 2.0 criteria. The primary endpoint was invasive disease-free survival (iDFS), defined as the time from curative breast cancer surgery to the first occurrence of invasive recurrence, distant metastasis, a second primary non-breast invasive cancer, or death from any cause. Recurrence-free survival (RFS) was defined as the time from curative surgery to the first documented recurrence, including local invasive recurrence, distant metastasis, contralateral breast cancer, or death from any cause. Overall survival (OS) was defined as the time from curative surgery to death from any cause. Patients without an event were censored at the data cutoff date (September 30, 2025) or the date of the last follow-up.

All efficacy analyses were conducted according to the initial postoperative treatment regimen assigned to each patient, regardless of any treatment interruptions or switches during the postoperative period. AEs were graded using the Common Terminology Criteria for Adverse Events (CTCAE) version 5.0.

### PSM

Given the non-random assignment of postoperative adjuvant therapy, propensity score matching (PSM) was employed to minimize potential confounding and selection bias. The propensity score model incorporated baseline clinicopathological characteristics likely to influence treatment allocation and prognosis, including age, molecular subtype, neoadjuvant chemotherapy regimen, neoadjuvant HER2-targeted therapy regimen, clinical stage before NAT, pathological stage after surgery, RCB class, and pathological nodal status. These covariates are listed in **Supplementary Table 1**. Patients were matched 1:1 using nearest-neighbor matching without replacement, with a caliper width of 0.2 times the standard deviation of the logit of the propensity score. Matching adequacy was evaluated by comparing standardized mean differences (SMDs) before and after matching, as well as the distribution of propensity scores between treatment groups.

### Statistical analysis

Continuous variables are presented as mean (SD) or median (interquartile range), while categorical variables are expressed as frequencies and percentages. Patients were divided into two groups according to their postoperative treatment regimen (HP or T-DM1). Baseline categorical variables were compared using the χ^2^ test or Fisher’s exact test, as appropriate. Ordinal variables were compared using the Mann-Whitney U test, and continuous variables were compared using the Student’s t-test or Welch’s t-test, as appropriate, following normality assessment with the Shapiro–Wilk test.

The Kaplan-Meier method was used to estimate 3-year survival rates, with differences between groups assessed using the log-rank test. Cox proportional hazards regression models were applied to identify prognostic factors associated with survival outcomes. Multicollinearity among covariates was checked before model construction, and variables exhibiting substantial collinearity were excluded to enhance model stability. Hazard ratios (HRs) and 95% confidence intervals (CIs) were calculated for each variable. Due to the relatively limited number of events, particularly for OS, Cox regression analyses focused primarily on iDFS and RFS, while OS analysis was considered exploratory. Sensitivity analyses were conducted by comparing treatment effects in both the unmatched original cohort and the PSM cohort to assess the robustness of the findings. All tests were two-sided, with a *P*-value < 0.05 considered statistically significant. Statistical analyses were performed using R software (version 4.3.1) and Python (version 3.9.23).

## Results

### Selection of the study population

**Figure 1:**
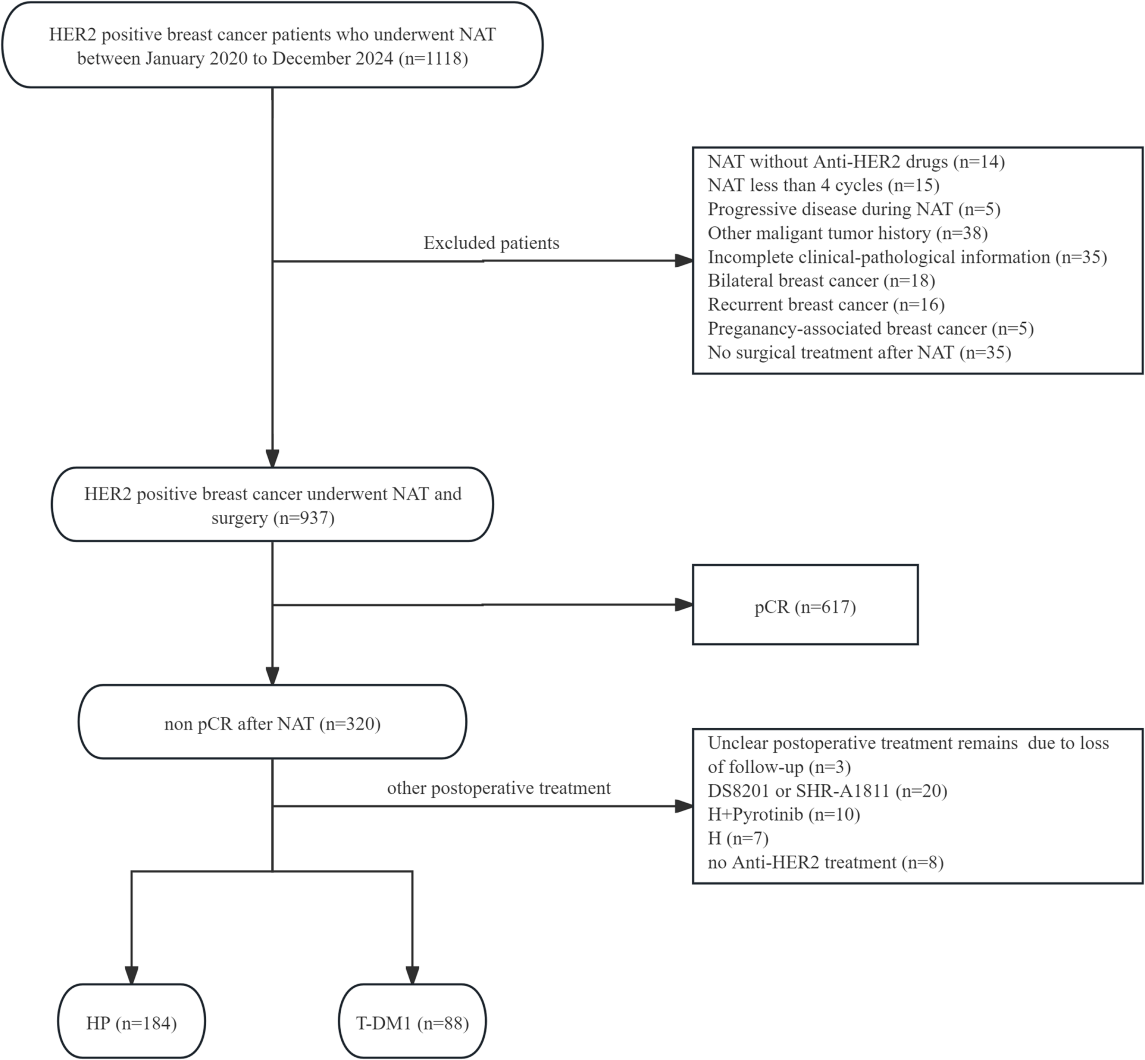
Flowchart of patients selection. A flowchart illustrating the patient selection process for this study. A total of 1,118 patients with HER2-positive breast cancer who underwent neoadjuvant therapy (NAT) were screened, of whom 937 met the eligibility criteria. After exclusion of pathological complete responders and individuals with unclear postoperative treatment information, 272 patients with residual invasive disease and definitive adjuvant therapy (HP or T-DM1) were included in the final analysis.

Between January 2020 and December 2024, a total of 1,118 patients with HER2-positive breast cancer received NAT at the Fourth Hospital of Hebei Medical University. Postoperative histopathological evaluation of paraffin-embedded specimens revealed that 320 patients did not achieve pCR, of which 272 received adjuvant therapy with either the HP regimen or the T-DM1 regimen (HP group: n = 184, T-DM1 group: n = 88). The details of the patient screening process are shown in **Figure 1**.

### Baseline characteristics before PSM

Detailed patient characteristics are presented in **Table 1**. All patients were female, with a median age of 52.5 years (range: 26.0–76.0). Significant baseline differences were observed between the two groups. Compared to patients in the HP group, those in the T-DM1 group were younger (median age: 50.0 vs. 53.5 years, *P* = 0.046) and exhibited a greater tumor burden, as evidenced by a higher proportion of ypN3 disease (*P* < 0.001), more advanced postoperative pathological stage (*P* = 0.003), and a higher proportion of RCB III lesions (*P* = 0.008). Additionally, with respect to NAT regimens, a higher proportion of patients in the T-DM1 group received carboplatin- or pyrotinib-containing regimens than those in the HP group, while anthracycline-based chemotherapy regimens and trastuzumab monotherapy were more frequently administered in the HP group (all *P* < 0.01). The two groups were generally comparable in terms of menopausal status, tumor location, clinical stage, and molecular subtype. These imbalances indicate that, in real-world clinical practice, T-DM1 was preferentially administered to patients with higher-risk residual disease.

**Table 1:** Baseline characteristics before PSM.

Variable	Total	HP	T-DM1	*P* value
	n=272	n=184	n=88	
Age, Median (range), years	52.5 (26.0, 76.0)	53.5 (26.0, 76.0)	50.0 (32.0, 71.0)	0.046
Menopausal status, n(%)				0.303
Postmenopausal	139 (51.1)	98 (53.3)	41 (46.6)	
Premenopausal	133 (48.9)	86 (46.7)	47 (53.4)	
Family history, n(%)				0.697
No	235 (86.4)	160 (87.0)	75 (85.2)	
Yes	37 (13.6)	24 (13.0)	13 (14.8)	
Location, n(%)				0.945
Left	143 (52.6)	97 (52.7)	46 (52.3)	
Right	129 (47.4)	87 (47.3)	42 (47.7)	
Lesion numbers, n(%)				0.050
Multifocal	56 (20.6)	44 (23.9)	12 (13.6)	
Unifocal	216 (79.4)	140 (76.1)	76 (86.4)	
molecular subtype, n(%)				0.056
HER2-positive	96 (35.3)	72 (39.1)	24 (27.3)	
Luminal B (HER2-positive)	176 (64.7)	112 (60.9)	64 (72.7)	
Operability, n (%)				0.284
Operable	170 (62.5)	111 (60.3)	59 (67.0)	
Non-operable	102 (37.5)	73 (39.7)	29 (33.0)	
Stage before NAT, n(%)				0.120
II	136 (50.0)	86 (46.7)	50 (56.8)	
III	136 (50.0)	98 (53.3)	38 (43.2)	
Chemotherapy, n(%)				0.007
Anthracyclines containing	51 (18.8)	44 (23.9)	7 (8.0)	
Carboplatin containing	137 (50.4)	87 (47.3)	50 (56.8)	
Paclitaxel monotherapy	84 (30.9)	53 (28.8)	31 (35.2)	
HER2 targeted therapy, n(%)				< 0.001
H	33 (12.1)	31 (16.8)	2 (2.3)	
HP	208 (76.5)	141 (76.6)	67 (76.1)	
HPy	31 (11.4)	12 (6.5)	19 (21.6)	
Surgery of breast, n(%)				0.609
Breast-conserving surgery	39 (14.3)	25 (13.6)	14 (15.9)	
Mastectomy	233 (85.7)	159 (86.4)	74 (84.1)	
Surgery of regional lymph nodes, n(%)				0.723
ALND	263 (96.7)	177 (96.2)	86 (97.7)	
SLNB	9 (3.3)	7 (3.8)	2 (2.3)	
Residual tumor size, Mean (SD), cm	1.3 (1.2)	1.2 (1.2)	1.3 (1.1)	0.379
Residual lymph grade, n(%)				< 0.001
ypN0	93 (34.2)	67 (36.4)	26 (29.5)	
ypN1	126 (46.3)	91 (49.5)	35 (39.8)	
ypN2	41 (15.1)	25 (13.6)	16 (18.2)	
ypN3	12 (4.4)	1 (0.5)	11 (12.5)	
stage after NAT, n(%)				0.003
I	103 (37.9)	73 (39.7)	30 (34.1)	
II	115 (42.3)	85 (46.2)	30 (34.1)	
III	54 (19.9)	26 (14.1)	28 (31.8)	
MP, n(%)				0.074
1	1 (0.4)	0 (0.0)	1 (1.1)	
2	17 (6.2)	11 (6.0)	6 (6.8)	
3	115 (42.3)	69 (37.5)	46 (52.3)	
4	90 (33.1)	67 (36.4)	23 (26.1)	
5	49 (18.0)	37 (20.1)	12 (13.6)	
RCB, n(%)				0.008
I	98 (36.0)	75 (40.8)	23 (26.1)	
II	120 (44.1)	81 (44.0)	39 (44.3)	
III	54 (19.9)	28 (15.2)	26 (29.5)	
bpCR, n(%)				0.230
bpCR	48 (17.6)	36 (19.6)	12 (13.6)	
non-bpCR	224 (82.4)	148 (80.4)	76 (86.4)	

H, trastuzumab; HP, trastuzumab plus pertuzumab; HPy, trastuzumab plus pyrotinib; T-DM1, trastuzumab emtansine; SLNB, sentinel lymph node biopsy; ALND, axillary lymph node dissection; MP, Miller-Payne grade; RCB, residual cancer burden; bpCR, breast pathological complete response.

### Efficacy analysis before PSM

After a median follow-up of 37.7 months (range, 9.2–68.7 months), 9 patients were lost to follow-up (7 in the HP group and 2 in the T-DM1 group). Disease recurrence occurred in 17 patients in the HP group (5 local recurrences and 12 distant metastases) and 10 patients in the T-DM1 group (3 local recurrences and 7 distant metastases). Breast cancer-specific death was observed in 11 patients, including 8 in the HP group and 3 in the T-DM1 group.

**Figure 2:**
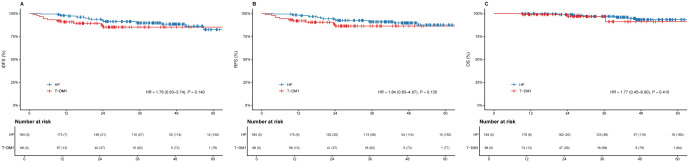
Kaplan–Meier survival curves before PSM. Kaplan-Meier survival curves for iDFS **(A)**, RFS **(B)**, OS **(C)** before PSM. iDFS, invasive disease-free survival; RFS, recurrence-free survival; OS, overall survival; HP, trastuzumab plus pertuzumab; T-DM1, trastuzumab emtansine; HR, hazard ratio.

Survival analysis did not detect statistically significant differences in short-term survival outcomes between the HP and T-DM1 groups before PSM. The 3-year iDFS rate was 89.70% (95% CI: 85.14%–94.50%) in the HP group and 85.18% (95% CI: 77.12%–94.08%) in the T-DM1 group, with no statistically significant difference observed (HR = 1.76; 95% CI: 0.83–3.74; *P* = 0.140, **Figure 2A**). Similarly, there were no significant differences between the two groups in 3-year RFS (90.92% vs. 86.36%; *P* = 0.135; **Figure 2B**) or OS (96.84% vs. 96.78%; *P* = 0.410; **Figure 2C**).

### Prognostic factors for survival outcomes

After adjusting for potential confounding factors using the Cox proportional hazards model, molecular subtype was identified as an independent prognostic factor for iDFS (HR = 0.43; 95% CI: 0.21–0.88; *P* = 0.022). Patients with the luminal B (HER2-positive) subtype had a significantly lower risk of iDFS compared to those with HER2-enriched breast cancer.

Molecular subtype and RCB grade were identified as independent prognostic factors for RFS. Specifically, multivariate analysis revealed that the HR-positive/HER2-positive subtype had a significant protective effect on RFS (HR = 0.34; 95% CI: 0.15–0.74; *P* = 0.007), whereas RCB grade III was an independent risk factor for disease recurrence (HR = 3.03; 95% CI: 1.06–8.67; *P* = 0.039). In contrast, no independent prognostic factors for OS were identified, likely due to the limited number of OS events. The complete univariate and multivariate Cox proportional hazards models are summarized in **Table 2**.

**Table 2:** Univariate and multivariate Cox analyses before PSM.

Variables	iDFS			
	Univariate Cox		Multivariate Cox	
	HR (95%CI)	*P* value	HR (95%CI)	*P* value
Age grade				
>40	Ref		Ref	
≤40	1.17 (0.48 ~ 2.85)	0.729	1.13 (0.46 ~ 2.76)	0.789
RCB				
I	Ref		Ref	
II	1.11 (0.48 ~ 2.61)	0.802	1.07 (0.45 ~ 2.50)	0.883
III	2.15 (0.85 ~ 5.43)	0.105	1.95 (0.76 ~ 5.01)	0.163
Molecular subtype				
HER2-positive	Ref		Ref	
Luminal B (HER2-positive)	0.50 (0.25 ~ 1.01)	0.053	0.43 (0.21 ~ 0.88)	0.022
Postoperative treatment regimen				
HP	Ref		Ref	
T-DM1	1.76 (0.83 ~ 3.74)	0.14	1.60 (0.74 ~ 3.46)	0.228

Variables	RFS			
	Univariate Cox		Multivariate Cox	
	HR (95%CI)	*P* value	HR (95%CI)	*P* value
Age grade				
>40	Ref		Ref	
≤40	1.14 (0.43 ~ 3.01)	0.794	1.09 (0.41 ~ 2.90)	0.855
RCB				
I	Ref		Ref	
II	1.58 (0.59 ~ 4.22)	0.358	1.52 (0.57 ~ 4.05)	0.407
III	3.33 (1.18 ~ 9.38)	0.023	3.03 (1.06 ~ 8.67)	0.039
Molecular subtype				
HER2-positive	Ref		Ref	
Luminal B (HER2-positive)	0.41 (0.19 ~ 0.89)	0.023	0.34 (0.15 ~ 0.74)	0.007
Postoperative treatment regimen				
HP	Ref		Ref	
T-DM1	1.84 (0.83 ~ 4.07)	0.135	1.59 (0.71 ~ 3.56)	0.265

Variables	OS			
	Univariate Cox		Multivariate Cox	
	HR (95%CI)	*P* value	HR (95%CI)	*P* value
Age grade				
>40	Ref		Ref	
≤40	1.77 (0.47 ~ 6.66)	0.401	1.74 (0.45 ~ 6.65)	0.421
RCB				
I	Ref		Ref	
II	1.03 (0.23 ~ 4.62)	0.965	0.97 (0.22 ~ 4.35)	0.967
III	3.40 (0.75 ~ 15.32)	0.112	3.15 (0.69 ~ 14.46)	0.139
Molecular subtype				
HER2-positive	Ref		Ref	
Luminal B (HER2-positive)	0.68 (0.21 ~ 2.23)	0.525	0.66 (0.19 ~ 2.27)	0.507
Postoperative treatment regimen				
HP	Ref		Ref	
T-DM1	1.77 (0.45 ~ 6.90)	0.41	1.45 (0.36 ~ 5.85)	0.601

iDFS, invasive disease-free survival; RFS, recurrence-free survival; OS, overall survival; HP, trastuzumab plus pertuzumab; T-DM1, trastuzumab emtansine; HR, Hazard ratio; Ref, Reference group.

### Efficacy analysis after PSM

The propensity score model showed acceptable discrimination for treatment assignment, with a c-statistic of 0.796. Among the 88 patients initially treated with T-DM1, 67 were successfully matched and 21 were unmatched and excluded, corresponding to 23.9% of the original T-DM1 cohort. The final matched cohort included 134 patients, with 67 patients in each treatment group. The median age of the matched cohort was 50.0 years (range: 26.0–71.0 years), and the median follow-up was 32.6 months (range: 9.2–67.8 months). Baseline characteristics were well balanced between the two groups, with no statistically significant differences (all *P* > 0.05, **Table 3**). The reliability of the matching process was further validated by the SMD plot (**Supplementary Figure 1**).

**Table 3:** Baseline characteristics after PSM.

Variable	Total	HP	T-DM1	*P* value
	n=134	n=67	n=67	
Age	50.0 (26.0, 71.0)	50.0 (26.0, 71.0)	50.0 (32.0, 69.0)	0.959
Menopausal status, n(%)				1.000
Postmenopausal	62 (46.3)	31 (46.3)	31 (46.3)	
Premenopausal	72 (53.7)	36 (53.7)	36 (53.7)	
Family history, n(%)				0.662
No	108 (80.6)	53 (79.1)	55 (82.1)	
Yes	26 (19.4)	14 (20.9)	12 (17.9)	
Location, n(%)				0.863
Left	71 (53.0)	36 (53.7)	35 (52.2)	
Right	63 (47.0)	31 (46.3)	32 (47.8)	
Lesion numbers, n(%)				0.492
Multifocal	23 (17.2)	13 (19.4)	10 (14.9)	
Unifocal	111 (82.8)	54 (80.6)	57 (85.1)	
molecular subtype, n(%)				0.568
HER2-positive	39 (29.1)	18 (26.9)	21 (31.3)	
LuminalB(HER2-pos)	95 (70.9)	49 (73.1)	46 (68.7)	
Operability, n (%)				1.000
Operable	90 (67.2)	45 (67.2)	45 (67.2)	
Non-operable	44 (32.8)	22 (32.8)	22 (32.8)	
Stage before NAT, n(%)				0.862
2	75 (56.0)	37 (55.2)	38 (56.7)	
3	59 (44.0)	30 (44.8)	29 (43.3)	
Chemotherapy, n(%)				0.347
Anthracyclines containing	12 (9.0)	5 (7.5)	7 (10.4)	
Carboplatin containing	88 (65.7)	48 (71.6)	40 (59.7)	
Paclitaxel monotherapy	34 (25.4)	14 (20.9)	20 (29.9)	
HER2 targeted therapy, n(%)				0.963
H	4 (3.0)	2 (3.0)	2 (3.0)	
HP	115 (85.8)	58 (86.6)	57 (85.1)	
HPy	15 (11.2)	7 (10.4)	8 (11.9)	
Surgery of breast, n(%)				0.612
Breast-conserving surgery	18 (13.4)	8 (11.9)	10 (14.9)	
Mastectomy	116 (86.6)	59 (88.1)	57 (85.1)	
Residual tumor size	1.3 (1.1)	1.3 (1.1)	1.3 (1.2)	0.916
Surgery of regional lymph nodes, n(%)				0.619
ALND	130 (97.0)	64 (95.5)	66 (98.5)	
SLNB	4 (3.0)	3 (4.5)	1 (1.5)	
Residual lymph grade, n(%)				0.114
ypN0	46 (34.3)	24 (35.8)	22 (32.8)	
ypN1mic~N1	65 (48.5)	34 (50.7)	31 (46.3)	
ypN2	14 (10.4)	8 (11.9)	6 (9.0)	
ypN3	9 (6.7)	1 (1.5)	8 (11.9)	
stage after NAT, n(%)				0.373
1	55 (41.0)	30 (44.8)	25 (37.3)	
2	55 (41.0)	28 (41.8)	27 (40.3)	
3	24 (17.9)	9 (13.4)	15 (22.4)	
MP, n(%)				0.511
1	1 (0.7)	0 (0.0)	1 (1.5)	
2	9 (6.7)	5 (7.5)	4 (6.0)	
3	56 (41.8)	24 (35.8)	32 (47.8)	
4	44 (32.8)	24 (35.8)	20 (29.9)	
5	24 (17.9)	14 (20.9)	10 (14.9)	
RCB, n(%)				0.413
I	48 (35.8)	27 (40.3)	21 (31.3)	
II	61 (45.5)	30 (44.8)	31 (46.3)	
III	25 (18.7)	10 (14.9)	15 (22.4)	
bpCR, n(%)				0.492
bpCR	23 (17.2)	13 (19.4)	10 (14.9)	
non-bpCR	111 (82.8)	54 (80.6)	57 (85.1)	

H, trastuzumab; HP, trastuzumab plus pertuzumab; HPy, trastuzumab plus pyrotinib; T-DM1, trastuzumab emtansine; SLNB, sentinel lymph node biopsy; ALND, axillary lymph node dissection; MP, Miller-Payne grade; RCB, residual cancer burden; bpCR, breast pathological complete response.

**Figure 3:**
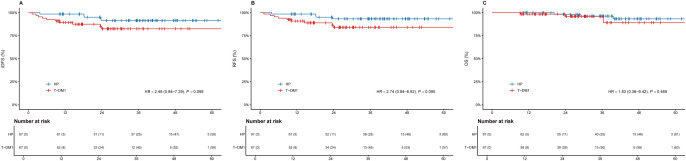
Kaplan–Meier survival curves after PSM. Kaplan-Meier survival curves for iDFS **(A)**, RFS **(B)**, OS **(C)** after PSM. iDFS, invasive disease-free survival; RFS, recurrence-free survival; OS, overall survival; HP, trastuzumab plus pertuzumab; T-DM1, trastuzumab emtansine; HR, hazard ratio.

**Figure 4:**
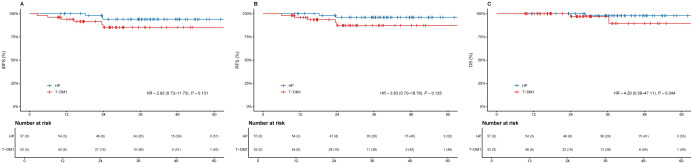
Forest Plot of Subgroup Analysis for iDFS, RFS, and OS After PSM. Forest plot showing the results of subgroup analysis for iDFS **(A)**, RFS **(B)**, OS **(C)** after PSM. HP, trastuzumab plus pertuzumab; T-DM1, trastuzumab emtansine; HR, hazard ratio.

The estimated 3-year iDFS rates after PSM were 91.52% in the HP group and 82.56% in the T-DM1 group. The corresponding 3-year RFS rates were 93.32% and 84.11%, respectively, and the 3-year OS rates were 96.31% and 96.02%, respectively. Survival analysis showed that the T-DM1 cohort did not exhibit significant survival advantages over the HP group in terms of iDFS (HR = 2.48; 95% CI: 0.84–7.29; *P* = 0.098), RFS (HR = 2.74; 95% CI: 0.84–8.92; *P* = 0.095), or OS (HR = 1.83; 95% CI: 0.36–9.42; *P* = 0.469; **Figures 3A–C**). Multivariate Cox regression analysis, adjusting for covariates (age, molecular subtype, and RCB class), confirmed that the adjuvant treatment regimen was not an independent predictor of iDFS or RFS (all *P* > 0.05, **Table 4**). However, RCB III was identified as a significant independent risk factor for both iDFS (HR = 7.64; 95% CI: 1.41–41.38; *P* = 0.018) and RFS (HR = 19.68; 95% CI: 2.15–179.84; *P* = 0.008). In contrast, Luminal B (HER2-positive) subtype was independently associated with improved RFS (HR = 0.24; 95% CI: 0.07–0.80; *P* = 0.019). Subgroup analyses, stratified by postoperative pathological stage, RCB class, and molecular subtype, were conducted to assess prognostic differences between the HP and T-DM1 cohorts. These analyses revealed no significant differences in iDFS, RFS, or OS between the two treatment regimens across any of the evaluated subgroups (all *P* > 0.05). A comprehensive summary of these subgroup analyses is presented in **Figure 4**.

**Table 4:** Univariate and multivariate Cox analyses after PSM.

Variables	iDFS			
	Univariate Cox		Multivariate Cox	
	HR (95% CI)	*P* value	HR (95% CI)	*P* value
Age grade				
>40	Ref		Ref	
≤40	0.29 (0.04–2.18)	0.227	0.47 (0.06–3.76)	0.478
RCB class				
I	Ref		Ref	
II	2.61 (0.54–12.58)	0.231	2.60 (0.52–12.95)	0.245
III	7.05 (1.42–34.97)	0.017	7.64 (1.41–41.38)	0.018
Molecular subtype				
HER2-positive	Ref		Ref	
Luminal B (HER2-positive)	0.53 (0.19–1.50)	0.232	0.39 (0.13–1.16)	0.090
Postoperative treatment regimenn				
HP	Ref		Ref	
T-DM1	2.48 (0.84–7.29)	0.098	2.04 (0.69–6.05)	0.200

Variables	RFS			
	Univariate Cox		Multivariate Cox	
	HR (95% CI)	P value	HR (95% CI)	P value
Age grade				
>40	Ref		Ref	
≤40	0.34 (0.04–2.61)	0.299	0.72 (0.09–5.98)	0.764
RCB class				
I	Ref		Ref	
II	4.58 (0.55–38.06)	0.159	5.00 (0.58–43.11)	0.143
III	14.44 (1.74–120.09)	0.014	19.68 (2.15–179.84)	0.008
Molecular subtype				
HER2-positive	Ref		Ref	
Luminal B (HER2-positive)	0.41 (0.14–1.21)	0.105	0.24 (0.07–0.80)	0.019
Postoperative treatment regimenn				
HP	Ref		Ref	
T-DM1	2.74 (0.84–8.92)	0.095	2.21 (0.67–7.29)	0.191

Variables	OS			
	Univariate Cox		Multivariate Cox	
	HR (95% CI)	P value	HR (95% CI)	P value
Age grade				
>40	Ref		Ref	
≤40	0.78 (0.09–6.71)	0.823	1.68 (0.16–17.71)	0.666
RCB class				
I	Ref		Ref	
II	NE (0.00–Inf)	0.999	NE (0.00–Inf)	0.999
III	NE (0.00–Inf)	0.999	NE (0.00–Inf)	0.998
Molecular subtype				
HER2-positive	Ref		Ref	
Luminal B (HER2-positive)	0.64 (0.12–3.53)	0.612	0.30 (0.04–1.95)	0.206
Postoperative treatment regimenn				
HP	Ref		Ref	
T-DM1	1.83 (0.36–9.42)	0.469	1.40 (0.26–7.54)	0.697

iDFS, invasive disease-free survival; RFS, recurrence-free survival; OS, overall survival; HP, trastuzumab plus pertuzumab; T-DM1, trastuzumab emtansine; HR, Hazard ratio; Ref, Reference group; NE, not estimable.

### Sensitivity analysis restricted to neoadjuvant dual HER2 blockade

To address the potential influence of neoadjuvant trastuzumab monotherapy, we performed an additional sensitivity analysis after excluding patients who had received neoadjuvant trastuzumab monotherapy. Specifically, 4 patients in the post-PSM cohort who had received neoadjuvant trastuzumab monotherapy were excluded. The final sensitivity analysis cohort included 130 patients, with 65 patients in each treatment group. The results were consistent with the main post-PSM analysis. No statistically significant differences were detected between adjuvant HP and T-DM1 in iDFS (HR = 2.51; 95% CI: 0.85–7.37; *P* = 0.095), RFS (HR = 2.76; 95% CI: 0.85–9.01; *P* = 0.092), or OS (HR = 1.88; 95% CI: 0.36–9.68; *P* = 0.452). The detailed results of this sensitivity analysis are shown in **Supplementary Table 2**.

### Exploratory analyses in clinically favorable subgroups

**Figure 5:**
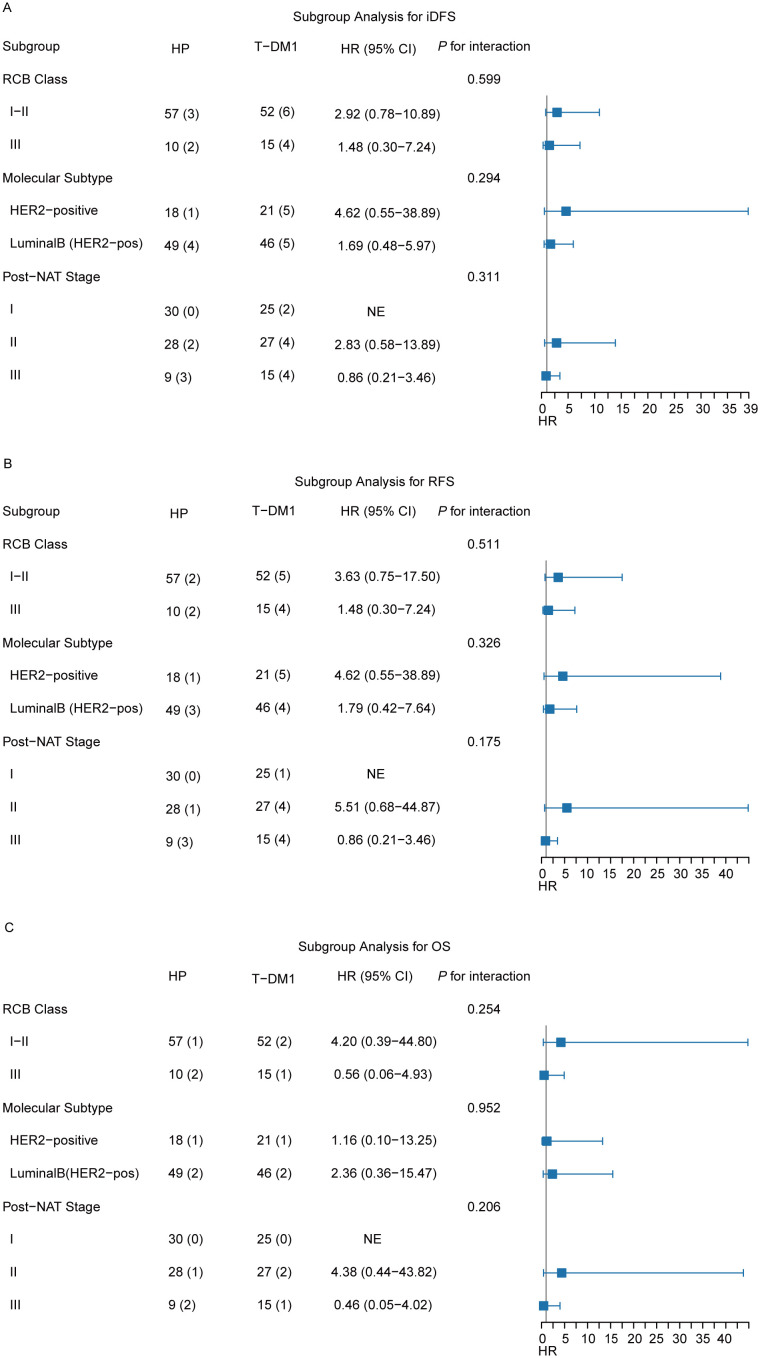
Kaplan–Meier survival curves in patients with Luminal B (HER2-positive) breast cancer. Kaplan–Meier survival curves for **(A)** iDFS, **(B)** RFS, and **(C)** OS among patients with Luminal B (HER2-positive) intrinsic subtype after NAT. HP, trastuzumab plus pertuzumab; T-DM1, trastuzumab emtansine; HR, hazard ratio.

**Figure 6:**
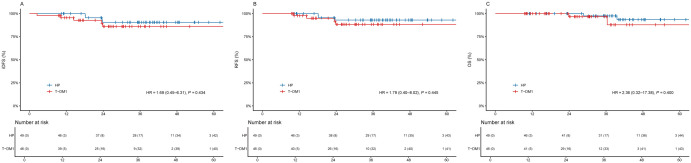
Kaplan–Meier survival curves in patients with low residual cancer burden (RCB I–II). Kaplan–Meier survival curves for **(A)** iDFS, **(B)** RFS, and **(C)** OS among patients with low residual cancer burden (RCB I–II). HP, trastuzumab plus pertuzumab; T-DM1, trastuzumab emtansine; HR, hazard ratio.

To further explore whether treatment effects varied across clinically favorable populations, survival analyses were conducted in patients with the luminal B (HER2-positive) subtype and in those with residual cancer burden (RCB) class I–II following NAT. Within the luminal B (HER2-positive) subgroup, Kaplan-Meier analysis revealed no significant differences between the two treatment arms in terms of iDFS (HR = 1.69; 95% CI: 0.45–6.31; *P* = 0.434), RFS (HR = 1.79; 95% CI: 0.40–8.02; *P* = 0.445), or OS (HR = 2.36; 95% CI: 0.32–17.38; *P* = 0.400) (**Figures 5A–C**). Similar results were observed among patients with RCB class I–II, in whom the choice between HP and T-DM1 did not result in statistically significant differences in iDFS (HR = 2.92; 95% CI: 0.73–11.73; *P* = 0.131), RFS (HR = 3.63; 95% CI: 0.70–18.76; *P* = 0.125), or OS (HR = 4.20; 95% CI: 0.38–47.11; *P* = 0.244) (**Figures 6A–C**). These subgroup findings should be interpreted as exploratory and hypothesis-generating.

### Adverse events before and after PSM

A comprehensive summary of treatment-related AEs before and after PSM is presented in **Table 5**. In the overall cohort, the global incidence of AEs was notably higher in the T-DM1 arm compared to the HP arm. As a result, treatment interruption or regimen modification was significantly more frequent in the T-DM1 group (22.7%, n = 20) than in the HP group (2.2%, n = 4). Treatment discontinuation in the HP cohort was due to arrhythmia (n = 2), decreased left ventricular ejection fraction (LVEF, n = 1), and financial constraints (n = 1). In the T-DM1 cohort, grade 3–4 AEs led to a regimen switch to HP (n = 12) or complete discontinuation (n = 4), while financial constraints caused interruptions in 4 patients. Patients without such events completed their planned 14-cycle T-DM1 or 1-year HP adjuvant regimens.

**Table 5:** Adverse events before and after PSM.

Variables	AEs before PSM				AEs after PSM			
	Total (n=272)	HP (n=184)	T-DM1 (n=88)	*P* value	Total (n=134)	HP (n=67)	T-DM1 (n=67)	*P* value
Discontinue or modification of treatment, n(%)				<0.001				<0.001
No	248 (91.2)	180 (97.8)	68 (77.3)		118 (88.1)	66 (98.5)	52 (77.6)	
Yes	24 (8.8)	4 (2.2)	20 (22.7)		16 (11.9)	1 (1.5)	15 (22.4)	
Decreased LVEF, n(%)				0.188				0.208
0	260 (95.6)	173 (94.0)	87 (98.9)		128 (95.5)	62 (92.5)	66 (98.5)	
1	11 (4.0)	10 (5.4)	1 (1.1)		6 (4.5)	5 (7.5)	1 (1.5)	
2	1 (0.4)	1 (0.5)	0 (0.0)					
Electrocardiogram abnormal, n(%)				0.308				
0	268 (98.5)	180 (97.8)	88 (100.0)		134 (100.0)	67 (100.0)	67 (100.0)	
1	4 (1.5)	4 (2.2)	0 (0.0)					
Thrombocytopenia, n(%)				<0.001				<0.001
0	226 (83.1)	184 (100.0)	42 (47.7)		98 (73.1)	67 (100.0)	31 (46.3)	
1	9 (3.3)	0 (0.0)	9 (10.2)		5 (3.7)	0 (0.0)	5 (7.5)	
2	11 (4.0)	0 (0.0)	11 (12.5)		10 (7.5)	0 (0.0)	10 (14.9)	
3	15 (5.5)	0 (0.0)	15 (17.0)		11 (8.2)	0 (0.0)	11 (16.4)	
4	11 (4.0)	0 (0.0)	11 (12.5)		10 (7.5)	0 (0.0)	10 (14.9)	
Neutropenia, n(%)				<0.001				0.014
0	261 (96.0)	184 (100.0)	77 (87.5)		126 (94.0)	67 (100.0)	59 (88.1)	
1	6 (2.2)	0 (0.0)	6 (6.8)		4 (3.0)	0 (0.0)	4 (6.0)	
2	5 (1.8)	0 (0.0)	5 (5.7)		4 (3.0)	0 (0.0)	4 (6.0)	
Anemia, n(%)				0.010				0.496
0	268 (98.5)	184 (100.0)	84 (95.5)		132 (98.5)	67 (100.0)	65 (97.0)	
1	4 (1.5)	0 (0.0)	4 (4.5)		2 (1.5)	0 (0.0)	2 (3.0)	
ALT or AST increased, n(%)				<0.001				<0.001
0	243 (89.3)	184 (100.0)	59 (67.0)		114 (85.1)	67 (100.0)	47 (70.1)	
1	21 (7.7)	0 (0.0)	21 (23.9)		14 (10.4)	0 (0.0)	14 (20.9)	
2	6 (2.2)	0 (0.0)	6 (6.8)		5 (3.7)	0 (0.0)	5 (7.5)	
3	2 (0.7)	0 (0.0)	2 (2.3)		1 (0.7)	0 (0.0)	1 (1.5)	
Interstitial lung disease, n(%)				0.122				0.362
0	270 (99.3)	184 (100.0)	86 (97.7)		132 (98.5)	67 (100.0)	65 (97.0)	
2	1 (0.4)	0 (0.0)	1 (1.1)		1 (0.7)	0 (0.0)	1 (1.5)	
4	1 (0.4)	0 (0.0)	1 (1.1)		1 (0.7)	0 (0.0)	1 (1.5)	
Mucositis oral, n(%)				0.324				1.000
0	271 (99.6)	184 (100.0)	87 (98.9)		133 (99.3)	67 (100.0)	66 (98.5)	
2	1 (0.4)	0 (0.0)	1 (1.1)		1 (0.7)	0 (0.0)	1 (1.5)	
Peripheral Neuropathy, n(%)				<0.001				0.008
0	259 (95.2)	184 (100.0)	75 (85.2)		125 (93.3)	67 (100.0)	58 (86.6)	
1	10 (3.7)	0 (0.0)	10 (11.4)		7 (5.2)	0 (0.0)	7 (10.4)	
2	3 (1.1)	0 (0.0)	3 (3.4)		2 (1.5)	0 (0.0)	2 (3.0)	
Conjunctivitis, n(%)				0.104				1.000
0	270 (99.3)	184 (100.0)	86 (97.7)		133 (99.3)	67 (100.0)	66 (98.5)	
1	2 (0.7)	0 (0.0)	2 (2.3)		1 (0.7)	0 (0.0)	1 (1.5)	
Rash, n(%)				0.218				0.222
0	269 (98.9)	182 (98.9)	87 (98.9)		131 (97.8)	65 (97.0)	66 (98.5)	
1	2 (0.7)	2 (1.1)	0 (0.0)		2 (1.5)	2 (3.0)	0 (0.0)	
2	1 (0.4)	0 (0.0)	1 (1.1)		1 (0.7)	0 (0.0)	1 (1.5)	
Vomiting/Diarrhea/Constipation, n(%)				0.290				0.604
0	264 (97.1)	180 (97.8)	84 (95.5)		131 (97.8)	66 (98.5)	65 (97.0)	
1	7 (2.6)	4 (2.2)	3 (3.4)		2 (1.5)	1 (1.5)	1 (1.5)	
4	1 (0.4)	0 (0.0)	1 (1.1)		1 (0.7)	0 (0.0)	1 (1.5)	

LVEF, left ventricular ejection fraction; ALT, alanine aminotransferase; AST, aspartate aminotransfer.

Specifically, the most common hematological AE in the T-DM1 arm was thrombocytopenia, with grade 3 or grade 4 incidences of 17.0% (n = 15) and 12.5% (n = 11), respectively. Neutropenia was the second most frequent hematological toxicity, though no grade 3–4 neutropenia or anemia was observed. For non-hematological toxicities, elevated transaminases (ALT/AST) were the most common (33.0%), followed by peripheral neuropathy (14.8%). Furthermore, two grade 4 non-hematological AEs occurred in the T-DM1 arm—one case of drug-related ILD and one case of acute intestinal obstruction—both of which required hospitalization and treatment discontinuation. In contrast, no grade 3–4 AEs were recorded in the HP arm.

After PSM, the distribution profile of AEs remained largely consistent with the overall cohort. The rate of treatment interruption or regimen modification in the matched T-DM1 group was 22.4% (n = 15), significantly higher than the 1.5% (n = 1) observed in the HP group. Notably, grade 3–4 thrombocytopenia remained the most frequent high-grade AE in the T-DM1 arm post-matching (31.3%, n = 21), while the HP arm continued to show no grade 3–4 AEs.

## Discussion

In this real-world retrospective study, no statistically significant difference in short-term survival outcomes was detected between adjuvant HP and T-DM1 among patients with HER2-positive residual invasive disease after NAT, while HP showed a more favorable safety and treatment-adherence profile; however, the wide confidence intervals preclude any conclusion of equivalent or non-inferior efficacy. These findings should therefore be interpreted as complementary and hypothesis-generating real-world evidence rather than as evidence to challenge the current standard role of T-DM1.

While the 3-year iDFS rate in the HP cohort of our study closely mirrors that reported in the APHINITY trial (6), the absolute iDFS rate observed in the T-DM1 cohort—especially following PSM—was lower than the efficacy outcomes demonstrated in the KATHERINE and DESTINY-Breast05 trials (4, 8). A major consideration in interpreting these findings is confounding by indication. Before PSM, patients receiving T-DM1 had a higher-risk disease profile, including a higher proportion of ypN3 disease, more advanced postoperative pathological stage, and more frequent RCB III disease. Although PSM improved the balance of measured baseline characteristics, numerically higher proportions of advanced nodal disease and RCB III remained in the T-DM1 group after matching, and residual confounding could not be completely excluded. In real-world practice, clinicians often prioritize T-DM1 for patients with higher residual tumor burden or more advanced pathological features, which may partly explain the numerically unfavorable HRs observed in the T-DM1 group after matching. Moreover, unmeasured biological factors, such as HER2 spatial heterogeneity, tumor-infiltrating lymphocytes, and intrinsic molecular subtypes, are not routinely captured in clinical records but may influence prognosis and treatment response (13, 14, 21–23). Therefore, our results should be interpreted as real-world comparative effectiveness and tolerability data rather than as evidence of causal efficacy differences between HP and T-DM1. These considerations also highlight the potential relevance of emerging genomic tools such as HER2DX, which integrates tumor biology with clinical risk factors and may further refine risk stratification in early-stage HER2-positive breast cancer (17–19).

Beyond efficacy, treatment safety and patient adherence are critical factors affecting real-world outcomes. In our cohort, the T-DM1 arm had a significantly higher incidence of grade ≥ 3 AEs—particularly thrombocytopenia—which necessitated treatment interruption or regimen modification in nearly a quarter of patients. Such early discontinuation, driven by the cumulative toxicity of ADCs, was similarly reported in a French national real-world cohort and may attenuate the expected clinical benefit of T-DM1 in routine practice (7). Additionally, existing literature and meta-analyses on HER2-targeted ADCs support the elevated risks of hematological toxicities, gastrointestinal events, and ILD/pneumonitis (24–27). In contrast, the HP regimen demonstrated a markedly superior safety profile in both our cohort and in historical trials. The APHINITY trial reported low incidences of high-grade toxicities and minimal treatment discontinuations (6), while the FeDeriCa and FDChina trials showed that fixed-dose subcutaneous HP formulations offer comparable safety and enhanced clinical convenience (9).

Multivariate Cox regression and subgroup analyses further emphasize the importance of precise risk stratification in guiding postoperative adjuvant treatment discussions. Intrinsic molecular subtype and RCB class were identified as key independent prognostic factors. Notably, within subgroups with more favorable prognoses—specifically the HR-positive/HER2-positive subtype and RCB I–II—no statistically significant differences in survival outcomes were detected between HP and T-DM1. Biologically, HR-positive/HER2-positive tumors exhibit dual-pathway dependency. Enhanced ER signaling may partially compensate for tumor reliance on the HER2 pathway, potentially reducing the efficacy of ADCs that depend on targeted endocytosis and intracellular payload delivery for cytotoxicity (28, 29). Moreover, in patients with minimal residual disease (RCB I–II) following NAT, surviving tumor cells may show lower HER2 expression density and slower proliferation rates, phenotypic changes that could limit ADC internalization and payload effect (30). These mechanistic considerations are hypothesis-supporting only and require prospective validation.

This study has several inherent limitations. First, due to its single-center retrospective design, selection bias cannot be entirely eliminated. Although rigorous PSM was employed to address observable confounders, institutional practice patterns may still influence the generalizability of our findings. Second, the modest sample size and low number of survival events limited the statistical power of the efficacy analyses, particularly after PSM. In the matched cohort, only 67 patients were included in each treatment group, and the HR estimates for iDFS, RFS, and OS had wide 95% CIs. Importantly, this study was retrospective and was not prospectively designed to test equivalence or non-inferiority, as no pre-specified non-inferiority margin was defined. Therefore, the absence of statistically significant differences should be interpreted only as a failure to detect a difference in this dataset, rather than as evidence of equivalent or non-inferior efficacy. Finally, our dataset did not allow for a granular comparison of outcomes between intravenous and subcutaneous HP formulations in this real-world setting.

In summary, our study highlights the marked heterogeneity of HER2-positive non-pCR disease after NAT and underscores the importance of individualized postoperative risk assessment. In this real-world cohort, no statistically significant survival difference was detected between HP and T-DM1, while HP showed a more favorable safety and treatment-adherence profile. Given the limited number of events and wide confidence intervals, these findings should be regarded as hypothesis-generating and should not be interpreted as evidence of equivalent or non-inferior efficacy.

## Conclusion

In this real-world cohort of patients with HER2-positive residual disease following NAT, no statistically significant difference in short-term recurrence or survival outcomes was detected between adjuvant HP and T-DM1. Exploratory subgroup analyses also did not show meaningful survival differences between the regimens. Toxicity profiles differed substantially: T-DM1 was associated with a significantly higher incidence of grade ≥ 3 AEs and subsequent treatment modifications, whereas HP demonstrated better tolerability and treatment adherence. These results provide complementary contemporary real-world evidence, but do not supplant the current standard role of T-DM1 and require prospective validation.

## Data Availability

The data analyzed in this study is subject to the following licenses/restrictions: The raw data supporting the conclusions of this article will be made available by the corresponding author on reasonable request. Requests to access these datasets should be directed to TZ, 47700364@hebmu.edu.cn.
