# Supplementary figures and images for: Comparative effectiveness and safety of adjuvant trastuzumab plus pertuzumab versus trastuzumab emtansine in HER2-positive breast cancer with residual disease after neoadjuvant therapy: a real-world retrospective study
Source: Front Oncol. 2026 Jun 26;16:1852055. doi: 10.3389/fonc.2026.1852055 (PMC13349879; doi:10.3389/fonc.2026.1852055)

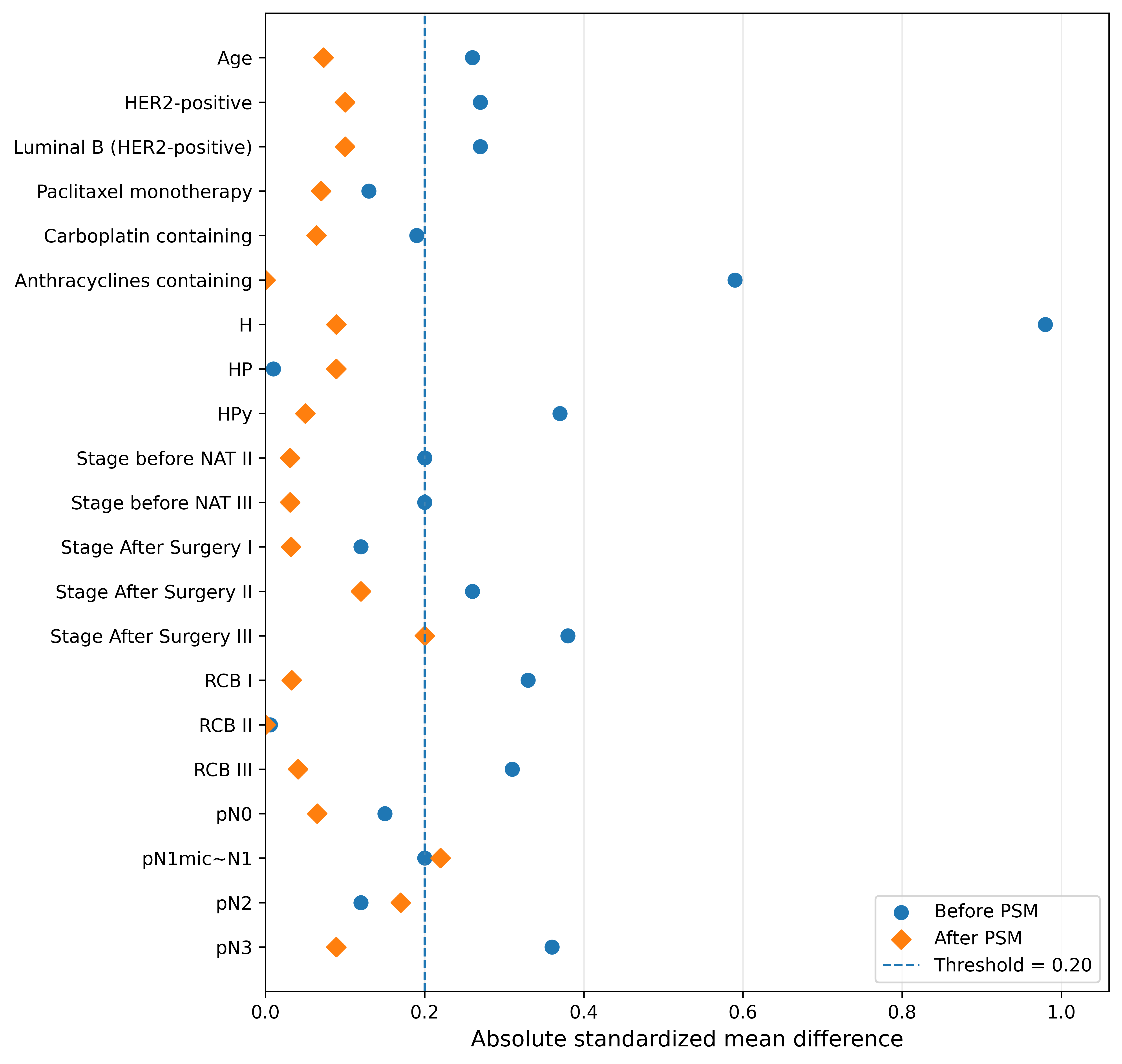

Supplement: Supplementary file 1 [file Image1.tiff]
