# Supplementary material for: Comparative effectiveness and safety of adjuvant trastuzumab plus pertuzumab versus trastuzumab emtansine in HER2-positive breast cancer with residual disease after neoadjuvant therapy: a real-world retrospective study
Source: Front Oncol. 2026 Jun 26;16:1852055. doi: 10.3389/fonc.2026.1852055 (PMC13349879; doi:10.3389/fonc.2026.1852055)
Supplement: Supplementary file 2 [file Table1.docx]

Supplementary Material

Supplementary table 1. Covariates included in the propensity score model

| Variable | SMD before PSM | SMD after PSM |
| --- | --- | --- |
| Age, median (range), years | -0.26 | 0.073 |
| Molecular Subtype, n (%) |  |  |
| HER2-positive | -0.27 | 0.1 |
| Luminal B (HER2-positive) | 0.27 | -0.1 |
| Chemotherapy, n (%) |  |  |
| Paclitaxel monotherapy | 0.13 | 0.07 |
| Carboplatin containing | 0.19 | -0.064 |
| Anthracyclines  containing | -0.59 | 0 |
| HER2-targeted therapy, n(%) |  |  |
| H | -0.98 | -0.089 |
| HP | -0.01 | 0.089 |
| HPy | 0.37 | -0.05 |
| Stage before NAT, n (%) |  |  |
| II | 0.20 | -0.031 |
| III | -0.20 | 0.031 |
| Stage After Surgery, n (%) |  |  |
| I | -0.12 | -0.032 |
| II | -0.26 | -0.12 |
| III | 0.38 | 0.20 |
| RCB, n (%) |  |  |
| I | -0.33 | -0.033 |
| II | 0.006 | 0 |
| III | 0.31 | 0.041 |
| Pathological node status, n (%) |  |  |
| pN0 | -0.15 | 0.065 |
| pN1mic~N1 | -0.20 | -0.22 |
| pN2 | 0.12 | 0.17 |
| pN3 | 0.36 | 0.089 |

Supplementary Table 2. Sensitivity analysis restricted to neoadjuvant dual HER2 blockade

| Outcome | Events in HP | Events in T-DM1 | 3-year rate in HP | 3-year rate in T-DM1 | HR (T-DM1 vs HP) | 95% CI | *P* value |
| --- | --- | --- | --- | --- | --- | --- | --- |
| iDFS | 5 | 10 | 91.22% | 81.88% | 2.51 | 0.85–7.37 | 0.095 |
| RFS | 4 | 9 | 93.08% | 83.49% | 2.76 | 0.85–9.01 | 0.092 |
| OS | 3 | 3 | 96.17% | 95.85% | 1.88 | 0.36–9.68 | 0.452 |

Abbreviations: iDFS, invasive disease-free survival; RFS, recurrence-free survival; OS, overall survival; HP, trastuzumab plus pertuzumab; T-DM1, trastuzumab emtansine; HR, hazard ratio; CI, confidence interval.

Supplementary Figure 1. Density plot of propensity score before and after PSM

The distributions show substantial improvement in covariate balance following PSM, indicating successful matching. HP, trastuzumab plus pertuzumab; T-DM1, trastuzumab emtansine.
